# Psychosocial safety climate as a predictor of work engagement, creativity, innovation, and work performance: A case study of software engineers

**DOI:** 10.3389/fpsyg.2023.1082283

**Published:** 2023-04-06

**Authors:** Amy Zadow, May Young Loh, Maureen Frances Dollard, Gro Ellen Mathisen, Bella Yantcheva

**Affiliations:** ^1^Psychosocial Safety Climate Global Observatory, Centre for Workplace Excellence, Justice and Society, University of South Australia, Adelaide, SA, Australia; ^2^School of Psychology, Faculty of Health and Medical Sciences, University of Adelaide, Adelaide, SA, Australia; ^3^Faculty of Social Sciences, University of Stavanger, Stavanger, Norway

**Keywords:** creativity, psychosocial safety climate, engagement, innovation, work performance, software engineers

## Abstract

**Introduction:**

Creativity is vital for competitive advantage within technological environments facing the fourth industrial revolution. However, existing research on creativity has rarely addressed how a climate beneficial for worker psychological health, a psychosocial safety climate (PSC), could additionally stimulate the growth of workplace creativity, innovation, and performance in digital environments.

**Method:**

To examine how individually perceived PSC influences subsequent work engagement promoting higher levels of computer-based radical and incremental creativity, innovation, and work performance, employees in a software engineering firm (*N* = 29, 86 observations) completed a weekly questionnaire for 4 consecutive weeks.

**Results:**

At the between-person level PSC was positively related to average future weekly individual fluctuations of creativity (radical and incremental), work engagement, and job performance. Additionally weekly work engagement was related to future creativity (radical and incremental). Work engagement also mediated the between-person relationship between PSC and future creativity (both radical and incremental). PSC did not predict innovation.

**Discussion:**

This study contributes to the theory on PSC, creativity, and work performance by elucidating the individual perceived PSC-creativity relationship and suggesting PSC systems as meaningful antecedents to digital work performance.

## Introduction

The fourth industrial revolution (Industry 4.0) has transformed the way organizations operate in terms of the growth of advanced automation and robotics, human-to-machine communication and the Internet of Things, artificial intelligence, machine learning, sensor technology, and data analytics (European Agency for Safety Health at Work, 2018). As these embedded dynamic technological environments replace traditional administrative work tasks, promoting workplace creativity is critical and will become the foundation of competitive advantage, accelerating economic, social, and technological development (Anderson et al., 2014; Acar et al., 2019; Glaveanu et al., 2019; Huang, 2019; Shute and Rahimi, 2021). Capitalizing on the idea that organizations can create conditions that influence how employees perform at work, we examine a new way for organizations to optimize employee creativity and innovation. By establishing a psychosocial safety climate (PSC) (Dollard and Bakker, 2010), an environment that prioritizes psychological health and safety, we expect that employees working in digital environments will increase their creativity and innovation, leading to improved work performance. The present study among software engineers fills a gap in current research by investigating how individually perceived PSC predicts future levels of digital creativity, innovation, and work performance.

We aim to contribute to the literature in three ways. First, we extend the PSC theoretical framework (Dollard and Bakker, 2010; Zadow and Dollard, 2016; Zadow et al., 2019; Loh et al., 2020; Dollard and Bailey, 2021) to examine the impact of a psychologically healthy work climate (i.e., high PSC) on workplace creativity and innovation. An extensive review has highlighted the role of contextual individual psychosocial work design factors such as job control, coworker support, customer trust, and positive leadership in promoting employees' innovation and creativity (Brattström et al., 2012; Zhou and Hoever, 2014, 2022). Yet, there is a need to discover if the root causes of these psychosocial work design factors (e.g., perceived organizational policies, practices, and procedures that constitute the climate for psychological health and safety, the PSC) also influence creativity. Shifting thinking to the climate determinants of workplace creativity, rather than the job design, provides information to determine where best to target intervention to improve creativity. While previous creativity research has examined the influence of psychological safety, which is the sense of security to take interpersonal risks in a workplace either as a collective or an individual phenomenon (Edmondson, 1999; Madjar and Ortiz-Walters, 2009; Edmondson and Lei, 2014; Zhou and Pan, 2015; Frazier et al., 2017; Newman et al., 2017), and the impact of perceived organizational support (POS), described as the extent to which an organization is perceived to value the employee's contribution and care about their needs (Eisenberger et al., 1986, 2019; Kurtessis et al., 2017; Kim et al., 2022), it has not examined the climate for psychological health and safety (PSC; Dollard et al., 2019). It is important to differentiate the distinctive contribution of PSC (Dollard and Bakker, 2010; Hall et al., 2010; Dollard, 2019) because it is a theoretically and empirically separate construct with PSC more strongly related to psychological health and employee stress prevention (Idris et al., 2012). Although PSC is a well-documented organizational climate for better workplace psychological health and organizational effectiveness (see Zadow and Dollard, 2016; Zadow et al., 2019; Loh et al., 2020), and some evidence suggests that high PSC is related to creativity (Oppert et al., 2022), no research has assessed the role of individual-perceived PSC perceptions on employees' future creativity and innovation.

Second, as the construct of creativity (i.e., the ideation) and innovation (i.e., the implementation) are closely related but contain some unique aspects, we studied both simultaneously in the current study. We further examined two types of creativity, namely radical and incremental creativity providing a comprehensive view of workplace creativity and innovation. Finally, answering a call for empirical and methodological advancement in the field of creativity, we introduce a new alternative theoretical climate perspective using a dynamic approach to examine how creativity develops over time using short weekly time lags or a “shortitudinal” research design (Dormann and Griffin, 2015) and combining longitudinal and multilevel research design to understand multilevel causal effects (Arellano and Bond, 1991).

## Creativity and innovation

Creativity and innovation are instrumental for organizational novelty and productivity (Cropley, 2015). Creativity in workplaces refers to the development of new and helpful ideas (Acar and Runco, 2015; Amabile and Pratt, 2016; van Knippenberg, 2017; van Knippenberg and Hirst, 2020). New ideas are defined as unique compared to other options that are currently available, while helpful ideas are those considered valuable within the workplace setting (Shalley et al., 2004). Madjar et al. (2011) extend this thinking by proposing that there are two types of creativity: radical and incremental. Radical creativity is defined as a range of ideas that differ significantly from an organization's existing procedures, while incremental creativity involves the modification of existing practices (Madjar et al., 2011). Innovation, alternatively, involves the implementation of creative ideas, processes, products, or procedures (West and Farr, 1990; Anderson et al., 2014; Hughes et al., 2018; Acar et al., 2019; Wang et al., 2019). Although some researchers argue that creativity and innovation are two interrelated concepts with overlapping conceptual boundaries and high correlations (Anderson et al., 2014; van Knippenberg, 2017; Acar et al., 2019), we posit that possessing creative ideas differ from the implementation of ideas, requiring the study of both concepts (Anderson et al., 2014; Hughes et al., 2018).

Ensuring that employees maximize their creative output requires attention to the antecedents of workplace creativity. The literature identifies several different antecedents of creativity covering individual characteristics (e.g., personality, Zhang et al., 2019; Li et al., 2020; individual resources constituting hope, efficacy, resilience, and optimism, Teng et al., 2020; Ghafoor and Haar, 2022), and leaders' influences (e.g., transformational leadership; Hughes et al., 2018; Al Harbi et al., 2019; Lee et al., 2020). Creativity and innovation researchers acknowledge that individuals within organizations do not operate in a vacuum highlighting the importance of understanding the contextual psychosocial influences. The extant literature shows that individual creativity and innovation at work are reduced by negative contextual variables such as workplace social interaction uncertainty or anxiousness and unmanageable job demands (Camacho and Paulus, 1995; Amabile et al., 1996; Goncalo et al., 2015; Probst et al., 2020) and increased when there are high levels of psychological safety - an employee feels safe to express their feelings at work (Liang et al., 2012; Tu et al., 2019; Oppert et al., 2022). These findings suggest that the psychological health of employees, influenced by their work environment, may be instrumental in digital workplace creative expression. From an organizational climate perspective, employees are organized into work groups and teams, affecting individual perceptions of climate and subsequent creative output (Zhu et al., 2018). Climates for creativity have been explored in previous research with a range of tools developed (Amabile et al., 1996; Mathisen and Einarsen, 2004; Hunter et al., 2006, 2007; Mathisen et al., 2006; West and Sacramento, 2012), and with an emphasis on climate factors that specifically stimulate or hamper creativity or innovation (e.g., support for innovation and distractions from creative work). The role of a climate for the support of psychological health, known as a PSC, has not been examined, which is a notable gap in creativity research.

## Psychosocial safety climate

Psychosocial safety climate describes the extent to which policies, practices, and procedures within an organization value and support psychological health (Dollard and Bakker, 2010). The PSC theoretical framework extends job design models of work stress and engagement (e.g., Demand-Control theory, Karasek, 1979; JD-R model, Demerouti et al., 2001) outlining organizational and managerial actions preceding the development of the positive work conditions leading to high levels of psychological health (Zadow and Dollard, 2016; Loh et al., 2020). The four domains of PSC include senior management support and commitment to psychological health, the priority of psychological health over productivity, the extent and effectiveness of organizational communication, and the participation and involvement of all stakeholders in relation to matters of psychological health and safety (for the PSC-4 and PSC-12 scales, refer to Hall et al., 2010; Dollard, 2019). PSC is typically considered the property of the organization and a climate construct that is assessed by aggregating individual perceptions within the organization or team (Zadow and Dollard, 2016; Dollard et al., 2019; Loh et al., 2020).

While research has identified that the constructs of psychological safety and POS increase creativity (for psychological safety, refer to Kark and Carmeli, 2009; Madjar and Ortiz-Walters, 2009; Kessel et al., 2012; Carmeli et al., 2013; Zhou and Pan, 2015; Agarwal and Farndale, 2017; for POS, refer to Yu and Frenkel, 2013; Zhang et al., 2016; Duan et al., 2020; Aldabbas et al., 2021), it is also important to understand the role of PSC. PSC is empirically and conceptually separate from psychological safety, which has a focus on perceived safety to engage in interpersonal behaviors influencing learning and performance (Edmondson, 1999; Edmondson and Lei, 2014; Frazier et al., 2017; Newman et al., 2017; Huang and Liu, 2022). Psychological safety reflects the extent to which an employee feels safe to take action or voice their opinion without embarrassment or experiencing undesirable consequences (Edmondson, 1999). Unlike PSC, the psychological safety model describes the quality of interpersonal interactions among the team members (and their team leaders). It does not explicitly refer to the higher level organizational policies, practices, and procedures for psychological health and safety. Alternatively, PSC is also theoretically and empirically separate from POS, which measures the extent to which an organization is perceived to value the employee's contribution and care about their social needs leading to reciprocation by employees through obligation and gratitude (Eisenberger et al., 1986, 2019; Kurtessis et al., 2017; Kim et al., 2022). Again, PSC is more specifically related to the systems and infrastructure including the policies, practices, and procedures for psychological health and safety. Empirically PSC has a stronger relationship with psychosocial job demands and resources, and psychological health symptoms than the psychological safety and POS measures (Idris et al., 2012). It is theorized that employees working in low PSC environments perceive that they lack the infrastructure and systems of operation, or the workplace policies, practices, and procedures, to protect their psychological health and safety, leading to increased levels of work stress and decreased resources for creativity, innovation, and performance. Evidence suggests that climates need to be measured and taken into account when reviewing individual workplace processes as they are a fundamental building block for interpreting and understanding phenomena in organizations and have a broad influence on individual behavior (Schulte et al., 2009; Ostroff et al., 2013; Schneider et al., 2017).

## The role of PSC in improving workplace performance and creativity

Using the overarching theoretical framework of PSC, we examine the relationship between PSC and work performance. PSC has been established as the key driver for adequate work resourcing, preceding task-level resources such as leader support, coworker support, and job control (Dollard and Bailey, 2014; Bailey et al., 2015; Dollard et al., 2019). In addition, PSC also works as a resource caravan passageway attracting and linking resources together, forming a repertoire of work resources for employees to access (Loh et al., 2018). As explained by the conservation of resources (COR) theory (Hobfoll, 1989, 2001), workers will experience stress when there is the threat of a loss of resources, a loss of resources, or a lack of resource gain following resource investment. COR theory predicts that when confronted by adverse work conditions such as a low level of protection and support of their psychological health (low PSC), individuals will strive to minimize net loss of resources seeking to obtain, protect, and maintain resource levels, restricting additional activities, which may be beneficial for performance (Zadow et al., 2017). On the other hand, in high PSC environments, workers feel that they are protected from psychological resource loss due to factors such as unmanageable workload, poor supervisor support, or limited job control and are not required to invest energy and resources managing perceived threats to their psychological health (Dollard and Bailey, 2021). As workers in high PSC contexts are not struggling to maintain depleted resources, they may be more robust in handling task-related pressure and more able to invest energy actively maintaining a task goal in memory, adopting effective cognitive search strategies, and judging and refining ideas leading to higher levels of work performance (Akinola et al., 2019). Initial work conducted by Idris et al. (2015) has identified a lagged effect of PSC on work performance *via* individual work engagement. We predict:

**Hypothesis 1**. Individual-perceived PSC T1 will positively relate to future weekly work performance.

The PSC theoretical framework may also be applied to creative processes. In a high PSC work environment with freedom from psychosocial risks, leading to additional individual resourcing for creative energy, workers will have the opportunity to invest in creative work. The dual pathway model of creativity suggests that creative output requires cognitive flexibility and persistence (Nijstad et al., 2010). Generating creative, innovative ideas requires strong executive functioning including working memory, inhibition, and fluid intelligence (Benedek et al., 2012; Said-Metwaly et al., 2020). The reduction of cognitive resources available may lead to the employment of simpler cognitive strategies like a narrow attentional focus leading to common, unoriginal ideas (Byron et al., 2010). Relatedly, studies have concluded that emotional exhaustion decreases cognitive resources needed to engage in creative behaviors (Han et al., 2017; Liu et al., 2020; Opoku et al., 2022) as well as other relevant creativity-facilitating conditions like positive affect (Wright and Hobfoll, 2004) and optimism (Tuckey and Neall, 2014). However, the effect of pressure on workers' creativity is still somewhat unclear and contested (Byron et al., 2010; Gutnick et al., 2012). While some studies demonstrate pressure to have detrimental effects on workers' creativity (e.g., Amabile et al., 1990; Oldham and Cummings, 1996; Shalley and Perry-Smith, 2001), other studies have found positive or curvilinear associations (e.g., Yuan and Zhou, 2008; Eisenberger and Aselage, 2009; Ohly and Fritz, 2010; Schmitt et al., 2012). The extent to which workers perceive a demand as inhibitive and draining of their resources, or challenging and motivating, may depend on their perceived ability to manage the situation (secondary appraisal, Lazarus and Folkman, 1984; LePine et al., 2004, 2005; Liu et al., 2022). In a high PSC context where they perceive sufficient resources are available to handle the pressure, the perception of manageability could be enhanced, in turn, increasing their cognitive capability to generate creative ideas.

Moreover, the positive relationship between PSC and creativity and innovation may be due to the social exchange process (Blau, 1964). Creative behavior can also be considered a reciprocal response to the feeling of having psychological health protected and valued by a leader or organization, reflecting a high level of PSC. Creativity can be conceptualized as an extra-role behavior that employees are likely to engage and invest their energy when they believe their organization supports them (Wayne et al., 1997; Rhoades and Eisenberger, 2002). When the organization shows its attentiveness to policies, practices, and procedures to protect psychological health, workers may increase their creative endeavors in response to favorable treatment from their organization. In support of these propositions, several studies have documented the positive effects of an organizational supportive climate on workers' creative and innovative behavior (e.g., Amabile et al., 1996; Anderson and West, 1998; Mathisen et al., 2008). However, whereas most of these studies so far emphasize the more specific phenomenon of creative climates encouraging creative or innovative behavior, the PSC specifically supports psychological health. Drawing upon the research evidence, this study draws a link between psychological health and the context that supports psychological health, precipitating future creativity. Thus, we predict that:

**Hypothesis 2**. Individual-perceived PSC T1 will positively relate to future weekly creativity (radical creativity, incremental creativity, and innovation).

## The mediating role of work engagement

Using the PSC theoretical framework, we propose that a high level of PSC relates to worker creativity and innovation through a psychological mechanism. Building on the previous insights of how positive emotions may broaden and expand an individual's cognitive abilities and thinking processes, we expect that work engagement will mediate the relationship between PSC and workplace creativity. Work engagement is described as a positive state of mind involving vigor, dedication, and absorption (Schaufeli and Bakker, 2004). Vigor involves high levels of mental energy and resilience even when difficulties arise, while dedication entails being heavily involved in work, experiencing enthusiasm, inspiration, and pride, and finally, absorption conceptualizes feeling engrossed in work with time passing quickly (Bakker et al., 2014). While a review of work engagement indicates that work engagement is relatively stable (Macey and Schneider, 2008), there is some evidence to suggest that there are short-term weekly fluctuations in work engagement within one person (Sonnentag, 2003; Bakker and Bal, 2010). Research evidence shows PSC as a strong predictor of work engagement (Law et al., 2011; Garrick et al., 2014; Idris et al., 2015; Afsharian et al., 2016). In a high PSC environment, there is high protection and value of psychological health, indicating that there are plenty of resources available to fulfill workers' innate needs for autonomy, relatedness, and competence and, in turn, improve workers' positive state of wellbeing which can be captured as work engagement (Idris et al., 2015). Consequently, Bakker et al. (2020) propose that feeling engaged can be related to the broaden-and-build theory (Fredrickson, 2001; Fredrickson and Losada, 2005), where the positive emotion associated with engagement broadens the thinking and activities of employees at work, who find the work interesting and absorbing, enabling them to think about novel alternatives to current work problems. Evidence also suggests that engaged employees are open to new ideas about how to improve and modify work processes and may be more motivated to perform creatively and persist with setbacks (Bakker et al., 2012, 2020; Bakker and Xanthopoulou, 2013; Demerouti et al., 2015; Koch et al., 2015; Eldor and Harpaz, 2016).

Taken together, these combined mechanisms suggest:

**Hypothesis 3**. Individual-perceived PSC T1 will be positively related to future weekly work engagement.
**Hypothesis 4**. Individual-perceived PSC T1 will indirectly and positively influence future weekly creativity (radical creativity, incremental creativity, and innovation) *via* work engagement.

Examining PSC as an antecedent to creativity and subsequent performance using the PSC theoretical framework may also identify new mechanisms to improve creativity and work performance. The creativity literature has not addressed how creativity and innovation efforts affect work performance in embedded dynamic technological environments. While it is anticipated that the creative process will be advantageous for work performance, it is clear that attention to creative tasks would need to be balanced with multiple ubiquitous and potentially mundane administrative tasks to reach a high level of job performance (Zhang and Bartol, 2010). Zhang and Bartol (2010) conducted a meta-analysis identifying that focusing on the creative process has a detrimental effect on other aspects of jobs impacting overall job performance. There is potential that PSC may mitigate this process. Again, we propose that individuals in high PSC environments, where senior management is supportive of psychological health and reduces psychosocial threats such as high work pressure and low social support, will have the discretionary personal resources to engage in creative thinking and also additional administrative activities to increase creative activities and work performance (as described in COR theory; Hobfoll, 1989). It is proposed that:

**Hypothesis 5**. Individual-perceived PSC T1 will positively influence future weekly creativity (radical creativity, incremental creativity and innovation, and subsequent weekly performance).

**Figure 1:**
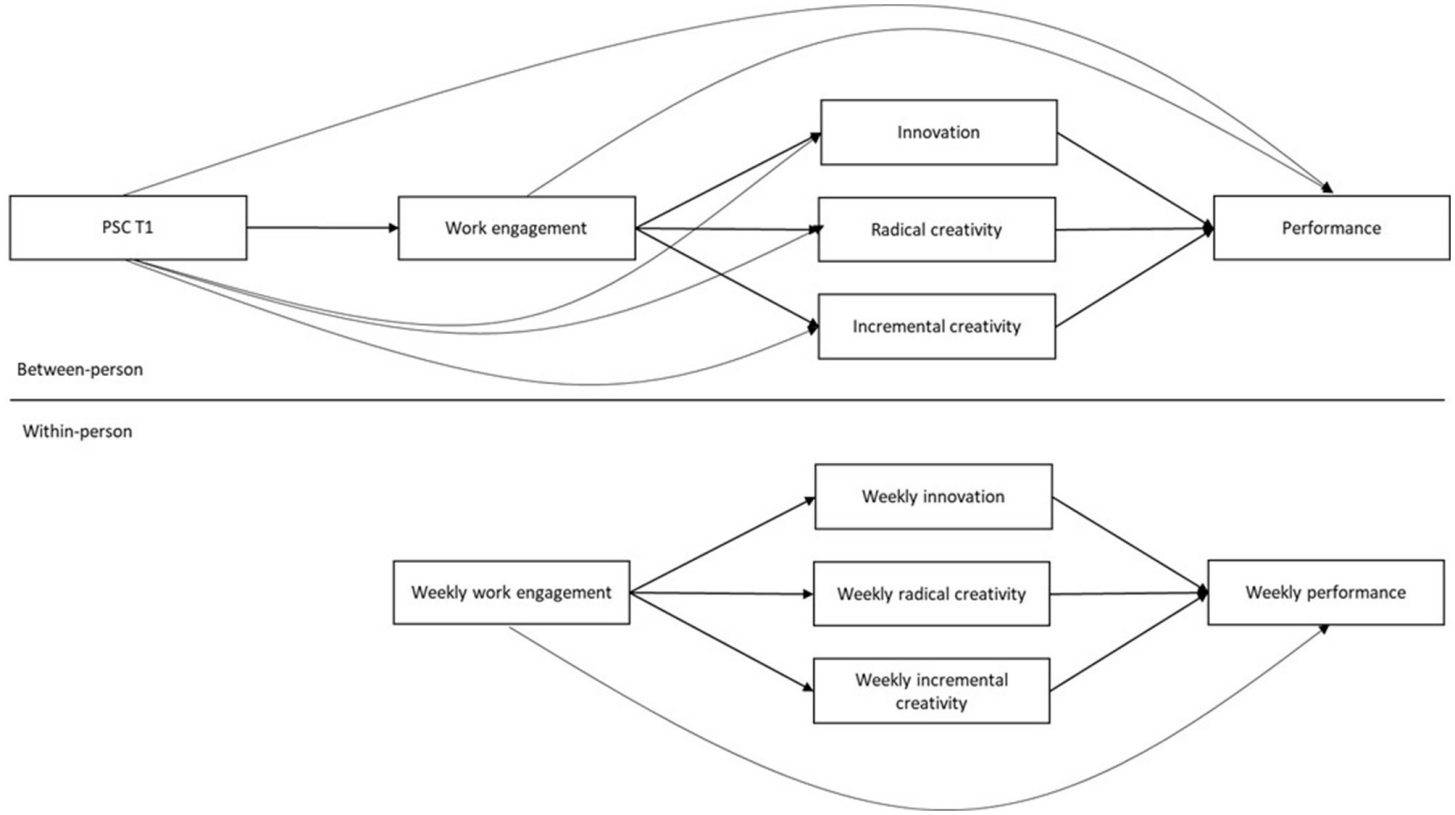
Hypothesized model.

The hypothesized model outlining the proposed hypotheses is provided in Figure 1. Figure 1 shows a two-level model with the between-person level measuring PSC and its impact on individual outcomes at the within-person level suggesting the pathway of weekly fluctuation of work engagement, creativity, innovation, and performance.

## Methods

### Participants and research design

A 4-week diary study was conducted between October to November 2019. Respondents were employees of a semiconductor technology company providing business services to engineering companies. The first author visited an Australian engineering firm and approached all the employees (*n* = 48) to invite them to participate in the survey. A total of 43 employees agreed to participate in the survey. The first author met them every week to complete the survey on an online platform. After matching the responses over time, the final sample included 29 individuals with 86 observations. Three participants in the sample were senior managers. All employees were university-qualified software engineers, and four had Ph.D. qualifications. Most of the participants (90%) were men. Five participants worked 50 h or more per week (17%). Ethics approval was obtained from the university of the authors, and participants provided informed consent to participate in the study (Protocol no.: 202172). The data supporting the findings of this study are available from the corresponding author, [AZ], upon reasonable request.

### Measures

The psychosocial safety climate was assessed using the PSC-12 scale (Hall et al., 2010). The PSC scale includes 12 questions which are categorized into four subscales. An example of an item is “Psychological wellbeing of staff is a priority for this organization.” Items are measured on a 5-point Likert scale, ranging from 1 (strongly disagree) to 5 (strongly agree).

Weekly work engagement was measured using The Utrecht Work Engagement Scale (UWES-9; Schaufeli et al., 2006). This shortened version scale with nine items consists of three subscales of engagement: (1) vigor, “At my work, I feel bursting with energy”; (2) dedication, “I am enthusiastic about my work”; and (3) absorption, “I am immersed in my work.” All items were measured on a 7-point Likert scale, ranging from 1 (never) to 7 (every day).

Weekly incremental creativity was measured using three items (Madjar et al., 2011). An example item is: “This week I have used previously existing ideas or work in an appropriate new way.” Items are measured on a 4-point Likert scale, ranging from 1 (never) to 4 (always).

Weekly radical creativity was measured using three items (Madjar et al., 2011). An example item is “This week I have suggested radically new ways for doing things.” Items are measured on a 4-point Likert scale, ranging from 1 (never) to 4 (always).

Weekly innovation was measured using items from the Eurofound 3rd European Company Survey 2013 Management Questionnaire. This was measured using four items. An example item is “This week, the work group contributed to new or significantly improved products or services.” Items are measured on a 7-point Likert scale, ranging from 1 (strongly disagree) to 7 (strongly agree).

Weekly performance was measured using 12 items (Williams and Anderson, 1991). Items included adequately completing assigned duties, fulfilling the responsibilities specified in job descriptions, performing expected tasks, meeting the formal performance requirements of the job, engaging in activities that will directly affect performance evaluations, and not neglecting aspects of the job an employee is obliged to perform and complete essential duties. Additional tasks over and above the job role were also measured including voluntarily doing more than was required and helping colleagues when they had too much work to do. An example item is “This week I have adequately completed assigned duties.” Items are measured on a 4-point Likert scale, ranging from 1 (strongly disagree) to 4 (strongly agree).

### Analysis procedure

The weekly diary data have a multilevel nature, with repeated measurements nested within the employees. Each respondent has four occasions. We, hence, conducted a multilevel model with the weekly repeated measures at level 1 (L1) and the individual at level 2 using the Hierarchical Linear Modeling (HLM) software. To avoid conflated estimates, we centered all the L1 predictors to the individual mean, and L2 predictors to the sample mean. At L1, we estimated within-person effects between the variables, while at L2, the between-person effects. To test the mediation process, we followed the suggestions of Preacher and Hayes (2004) by first examining the relationship between the predictor and mediators (X → M), followed by the relationship between mediators and outcome (M → Y). To confirm the significance of the mediational pathways, we ran the Monte Carlo simulations with 20,000 repetitions at a 95% confidence interval. We did not control for demographics or work status (i.e., position or tenure) as the information was kept confidential. Notably, previous scholars have found that gender and age do not exert strong influences on these estimates (Bernerth and Aguinis, 2016), recommending that demographic control variables should only be used when there is a strong theoretical hypothesis supporting their influence on participants' responses (Spector and Brannick, 2011). Self-reported measures of creativity and innovation were recommended by the Chief Executive Officer and Senior Managers of each work group who felt strongly, given the dynamic complexity of individual day-to-day work across multiple projects, that self-reports would be more accurate than manager ratings or group project outcomes. The managers also preferred the measurement of creativity and innovation in a real-life setting as they did not believe that role plays or construed activities could accurately reflect the fluctuating demands of multiple simultaneous projects experienced by software engineers.

### *Post-hoc* analysis

In addition to the mediation tests, we examined whether the level-1 slopes depicting the relationship between work engagement and creativity and between creativity and performance randomly vary between persons. Results showed that there is no adequate slope variance for an interaction relationship. But we also noted that this variance test is conservative and proceeded with moderation tests (Aguinis et al., 2013; Bliese et al., 2018). We ran the tests with PSC as the between-person moderator of the abovementioned relationships. All results showed that PSC did not moderate the relationships.

## Results

Table 1 shows the descriptives, reliabilities of the scales, and the within-person variance of the within-person constructs. Apart from PSC, which was only measured at week 1, we reported the range of internal consistency of the scale across 3 weeks from weeks 2 to 4.

**Table 1:** Means, standard deviations, reliability, bivariate correlations, and within-person variance.

	**Mean**	**SD**	**Cronbach alpha**	**1**	**2**	**3**	**4**	**5**	**6**	**Within-person variance**
Psychosocial safety climate Week 1	36.72	9.31	0.96	–						
Weekly work engagement	32.03	6.74	0.90–0.92	0.73^**^	–					0.86
Weekly radical creativity	8.44	2.63	0.80–0.83	0.55^**^	0.59^**^	–				0.75
Weekly incremental creativity	10.19	2.51	0.83–0.85	0.59^**^	0.70^**^	0.62^**^	–			0.80
Weekly innovation	15.95	5.16	0.90–0.94	0.36^**^	0.39^**^	0.53^**^	0.50^**^	–		0.77
Weekly performance	40.37	5.49	0.85–0.94	0.64^**^	0.67^**^	0.26^*^	0.57^**^	0.23^*^	–	0.80

Weekly data was averaged across Week 2 to Week 4. *N*_observation_ = 86; *N*_individual_ = 29. SD = standard deviation.

* *p* < 0.05;

** *p* < 0.01.

### Within-person variances for week-level variables

To justify the nature of the within-person constructs and the appropriateness of using multilevel analysis, we analyzed the within-person variance for each weekly variable. As opposed to the conventional intra-class correlation, we measured within-person variance by calculating the proportion of L1 variance as compared to the total variance (σ2/[σ2+τ00]) (Podsakoff et al., 2019). We found a substantial amount of within-person variance for each variable (86% for work engagement, 75% for radical creativity, 80% for incremental creativity, 77% for innovation, and 80% for work performance), following the benchmark reported by Podsakoff et al. (2019). But it is noteworthy that the benchmark was based on daily within-person variance, while this article uses weekly diary data. It is expected that the within-person variances would be higher from week to week as compared to a daily basis.

### The cross-level lagged effect of PSC Time 1 on the outcomes

Table 2 shows the results of PSC T1 on week-level outcomes. Hypothesis 1 stated that PSC T1 could positively predict performance. We first tested the cross-level lagged effect between PSC Week 1 at L2 and Work Performance at L1 (averaged across weeks 2–4). We controlled for the individual work performance in week 1. Results showed that the individual-perceived PSC at week 1 predicts the future work performance (averaged across weeks 2–4) of individuals (γ = 0.16, SE = 0.05, *t* = 3.07, *p* < 0.01). H1 is supported.

**Table 2:** HLM results of the effect of PSC on all the other variables, controlling baseline value.

	**Work engagement**			**Radical creativity**			**Incremental creativity**			**Innovation**			**Performance**		
	**Est**.	**SE**	***t*** **-value**	**Est**.	**SE**	***t*** **-value**	**Est**.	**SE**	***t*** **-value**	**Est**.	**SE**	***t*** **-value**	**Est**.	**SE**	***t*** **-value**
**Level 1**															
Intercept	30.51	0.79	38.68^***^	7.86	0.17	44.74^***^	9.65	0.30	31.82^***^	15.09	0.49	30.67^***^	36.89	0.69	53.63^***^
**Level 2**															
Work engagement T1	0.33	0.18	1.80^+^												
Radical creativity T1				0.61	0.07	8.41^***^									
Incremental creativity T1							0.38	0.12	3.19^**^						
Innovation T1										0.81	0.12	6.84^***^			
Performance T1													0.58	0.10	5.86^***^
PSC T1	0.39	0.10	3.74^**^	0.12	0.02	6.63^***^	0.13	0.02	5.09^***^	0.04	0.06	0.58	0.16	0.05	3.07^**^
L1 variance component	6.56			1.97			1.41			5.22			6.37		
L2 variance component	11.75			0.16			1.83			5.32			4.52		
Deviance	427.22			286.81			297.03			396.75			368.54		
Number of parameters	5			5			5			5			5		

*N*_observation_ = 86; *N*_individual_ = 29. PSC, psychosocial safety climate; Est., estimate. ^+^*p* < 0.10;

* *p* < 0.05;

** *p* < 0.01;

*** *p* < 0.001.

Hypothesis 2 predicted that PSC T1 would positively relate to future radical creativity, incremental creativity, and innovation (averaged across weeks 2–4). We ran three models separately. Again, we controlled for the individual-level radical creativity, incremental creativity, and innovation at T1. Results showed that PSC predicts radical creativity (γ = 0.12, SE = 0.02, *t* = 6.63, *p* < 0.001), incremental creativity (γ = 0.13, SE = 0.02, *t* = 5.09, *p* < 0.001), but not innovation (γ = 0.04, SE = 0.06, *t* = 0.58, not significant [ns], refer to Table 2). H2a and H2b are supported but not H2c.

Hypothesis 3 suggested that PSC T1 is positively related to future work engagement (averaged across weeks 2–4). Results showed that PSC T1 predicts work engagement (γ = 0.39, SE = 0.10, *t* = 3.74, *p* < 0.01). This supports H3.

### The indirect effect of PSC on work performance and creativity

After confirming the first and second conditions, we continued the analysis by running the models, which included both predictor and moderators, to test Hypotheses 4 and 5 (refer to Tables 3, 4).

**Table 3:** HLM results of weekly performance as an outcome.

**Model outcomes**	**Null model performance**			**Model 1a performance**			**Model 1b performance**			**Model 2 performance**		
	**Est**.	**SE**	***t*** **-value**	**Est**.	**SE**	***t*** **-value**	**Est**.	**SE**	***t*** **-value**	**Est**.	**SE**	***t*** **-value**
**Level 1**												
Intercept	36.22	0.81	44.96^***^	37.26	0.81	46.04^***^	37.30	0.70	53.40^***^	36.04	0.88	40.86^***^
Weekly work engagement				0.23	0.15	1.56	0.23	0.14	1.57			
Weekly radical creativity										0.37	0.33	1.11
Level 2												
PSC T1							0.10	0.10	1.03			
Performance T1	0.76	0.09	8.64^***^	0.57	0.13	4.26^***^	0.53	0.11	4.68^***^	0.79	0.10	7.94^***^
Work engagement				0.24	0.13	1.80^+^	0.15	0.19	0.79			
Radical creativity										−0.16	0.22	−0.74
L1 variance component	6.40			6.01			6.00			6.18		
L2 variance component	5.78			4.64			4.31			5.68		
Deviance	373.10			365.68			364.35			370.87		
Δ-2 log likelihood				7.42^*^			1.33			2.23		
Compared model				Null model			Model 1a			Null model		
Number of parameters	4			6			7			6		

*N*_observation_ = 86; *N*_individual_ = 29. PSC, psychosocial safety climate; Est., estimate. ^+^*p* < 0.10;

* *p* < 0.05;

** *p* < 0.01;

*** *p* < 0.001.

**Table 4:** HLM results of weekly radical and incremental creativity as outcomes.

**Model outcomes**	**Model 3a performance**			**Model 3b performance**			**Null model radical creativity**			**Model 4a radical creativity**			**Model 4b radical creativity**		
	**Est**.	**SE**	***t*** **-value**	**Est**.	**SE**	***t*** **-value**	**Est**.	**SE**	***t*** **-value**	**Est**.	**SE**	***t*** **-value**	**Est**.	**SE**	***t*** **-value**
**Level 1**															
Intercept	36.46	0.92	39.36^***^	36.74	0.74	49.70^***^	8.10	0.26	30.48^***^	8.14	0.20	40.19^***^	7.94	0.18	43.66^***^
Weekly work engagement										0.26	0.09	2.76^**^	0.26	0.09	2.76^**^
Weekly incremental creativity	1.07	0.17	6.39^**^	1.07	0.17	6.39^**^									
**Level 2**															
PSC T1				0.18	0.06	2.91^**^							0.09	0.02	4.01^***^
Performance T1	0.71	0.12	5.77^**^	0.61	0.11	5.54^**^									
Radical creativity T1							0.70	0.11	6.58^***^	0.59	0.08	7.25^***^	0.58	0.07	8.24^***^
Work engagement										0.16	0.03	4.49^***^	0.06	0.03	2.08^*^
Incremental creativity	0.17	0.29	0.57	−0.17	0.30	−0.56									
L1 variance component	4.72			4.71			1.97			1.51			1.51		
L2 variance component	6.29			4.98			1.27			0.52			0.26		
Deviance	358.27			353.66			310.38			279.07			270.97		
Δ−2 log likelihood	14.82^***^			4.60^*^						31.31^***^			8.09^***^		
Compared model	Null model			Model 3a						Null model			Model 4a		
Number of parameters	6			7			4			6			7		

*N*_observation_ = 86; *N*_individual_ = 29. PSC, psychosocial safety climate; Est., estimate. ^+^*p* < 0.10;

* *p* < 0.05;

** *p* < 0.01;

*** *p* < 0.001.

Hypothesis 4 stated that PSC T1 would indirectly and positively influence creativity and innovation *via* work engagement. However, as noted earlier, as the relationship between PSC and innovation did not show statistical significance, we excluded innovation in the following test. We first ran a null model for each creativity variable, controlling for the individual baseline score at the person level, and then tested the effect of work engagement on the variable. The results (refer to Tables 4, 5) showed that at the within-person level, weekly work engagement is only related to weekly radical creativity (γ = 0.26, SE = 0.09, *t* = 2.76, *p* < 0.01) but not incremental creativity (γ = 0.10, SE = 0.06, *t* = 1.60, ns). At the between-person level, work engagement is related to both radical creativity (γ = 0.06, SE = 0.03, *t* = 4.49, *p* < 0.05) and incremental creativity (γ = 0.21, SE = 0.04, *t* = 6.17, *p* < 0.001). To test the indirect effect, we ran a Monte Carlo simulation. The results (Table 6) confirmed that work engagement mediates the between-person relationship between PSC and future radical creativity (Lower level [LL] = 0.0003; Upper level [UL] = 0.0472, 95% confidence interval [CI]) and between PSC and incremental creativity (LL = 0.0497; UL = 0.1165, 95% CI). This supports Hypotheses 4a and 4b.

**Table 5:** HLM results of weekly incremental creativity and innovation as outcomes.

**Model outcomes**	**Null model incremental creativity**			**Model 5a incremental creativity**			**Model 5b incremental creativity**			**Null model innovation**			**Model 6a innovation**		
	**Est**.	**SE**	***t*** **-value**	**Est**.	**SE**	***t*** **-value**	**Est**.	**SE**	***t*** **-value**	**Est**.	**SE**	***t*** **-value**	**Est**.	**SE**	***t*** **-value**
**Level 1**															
Intercept	9.86	0.40	24.65^***^	9.98	0.25	39.72^***^	9.93	0.24	40.63^***^	15.15	0.53	28.36^***^	15.20	0.53	28.60^***^
Weekly work engagement				0.10	0.06	1.60	0.10	0.06	1.60				0.10	0.08	1.28
**Level 2**															
PSC T1							0.02	0.04	0.62						
Incremental creativity T1	0.54	0.16	3.46^**^	0.32	0.11	3.05^**^	0.31	0.11	2.97^**^						
Innovation T1										0.84	0.08	9.77^***^	0.77	0.12	6.98^***^
Work engagement				0.24	0.04	6.17^***^	0.21	0.04	4.65^***^				0.15	0.10	1.60
L1 variance component	1.41			1.34			1.34			5.22			5.14		
L2 variance component	3.16			1.16			1.14			5.41			4.59		
Deviance	309.49			284.95			284.61			397.13			392.90		
Δ-2 log likelihood				24.52^***^			0.35						4.23		
Compared model				Null model			Model 5a						Null model		
Number of parameters	4			6			7			4			6		

*N*_observation_ = 86; *N*_individual_ = 29. PSC, psychosocial safety climate; Est., estimate. ^+^*p* < 0.10;

* *p* < 0.05;

** *p* < 0.01;

*** *p* < 0.001.

**Table 6:** Monte Carlo simulation results for the indirect effect.

**Indirect effect**	**Confidence interval**	
	**Lower level**	**Upper level**
PSC → Work engagement → Performance	−0.0894	0.2053
PSC → Work engagement → Radical creativity	0.0003	0.0472
PSC → Work engagement → Incremental creativity	0.0497	0.1165

**Figure 2:**
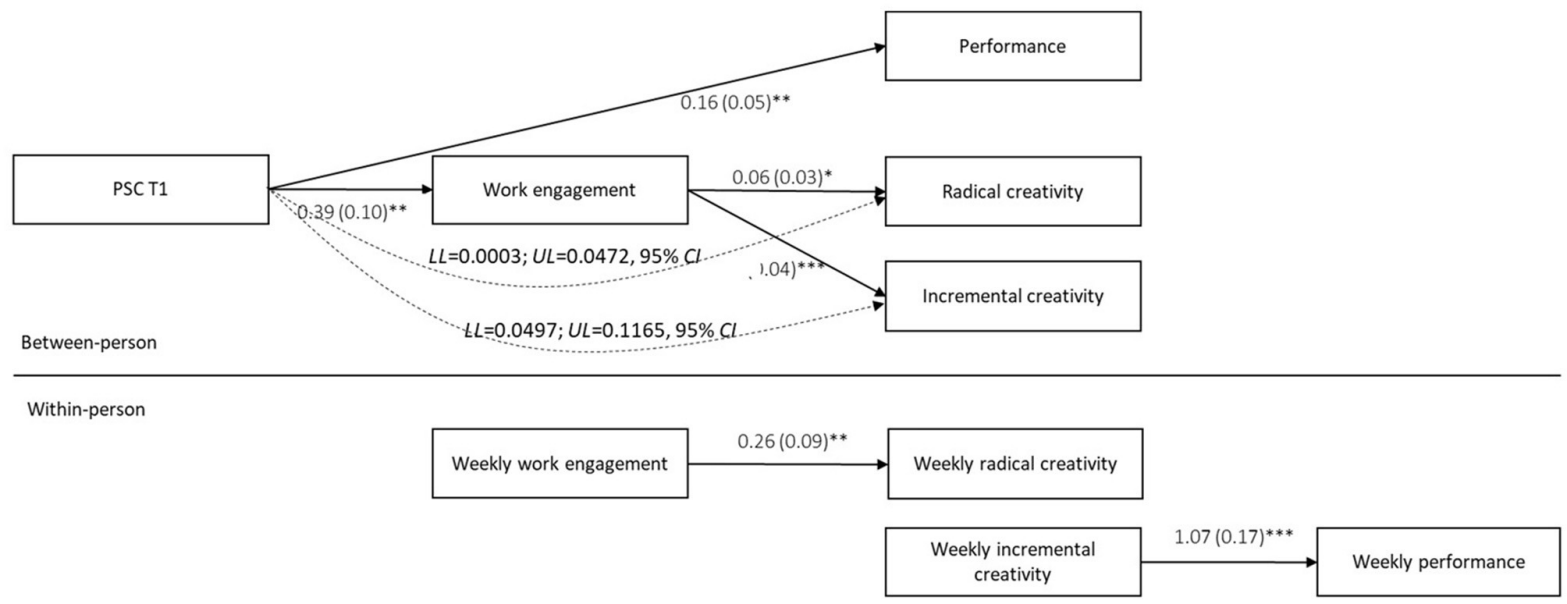
Results model. ^*^p < 0.05; ^**^p < 0.01; ^***^p < 0.001.

Hypothesis 5 suggested that PSC T1 would positively influence future creativity and, in turn, lead to improved work performance (refer to Table 4). As followed by the previous results, we excluded innovation due to the statistically insignificant relationship between PSC and innovation. We found weekly incremental creativity (γ = 1.07, SE = 0.17, *t* = 6.39, *p* < 0.001) but not weekly radical creativity (γ = 0.37, SE = 0.33, *t* = 1.11, ns) is related to weekly work performance. At the between-person level, both incremental and radical creativity are not related to work performance (incremental creativity: γ = 0.17, SE = 0.29, *t* = 0.57, ns; radical creativity: γ = −0.16, SE = 0.22, *t* = −0.74, ns). Hypothesis 5 is hence rejected. The results are shown in Figure 2.

## Discussion

Capitalizing on the idea that organizations can create climates that influence how individual employees think and perform at work, we examine a new way for organizations to optimize employee creativity in computer-based work. We applied a new theoretical framework, PSC theory, proposing that the climate for psychological health influences the development of future workplace creativity and job performance in the dynamic digital work environments of software engineers.

We found that individual-perceived PSC predicted future work performance and creativity (radical and incremental). Individual-perceived PSC was also positively related to future work engagement and weekly work engagement was related to future creativity (radical and incremental). Work engagement mediated the between-person relationship between PSC and future creativity (radical and incremental). This contribution is important because it suggests that there is a top-down impact of PSC impacting levels of creativity directly and *via* work engagement across time. The findings provide additional insights into the reasons why some employees may produce creative work. PSC may influence creative output in three ways. First, perceived high PSC may provide freedom from psychosocial risks leading to additional individual resourcing for creative energy. Second, a high PSC work environment where psychological health is valued and protected may encourage employees to reciprocate by completing additional creative activities. Third, PSC increases engagement and positive emotion, which may broaden the thinking and activities of employees who find the work interesting and absorbing, precipitating creative thought and practices. These findings suggest new contextual mechanisms for the etiology of workplace creativity, adding to existing theoretical frameworks which have identified that creative output results from security to take interpersonal risks (psychological safety) as a process of reciprocation when an employee feels valued and supported (POS) or through motivational processes and climates specifically targeting creativity (Mathisen and Einarsen, 2004; Mathisen et al., 2004, 2008; West and Richter, 2008; Liu et al., 2016; Sung et al., 2018; Fischer et al., 2019).

The psychosocial safety climate was not related to future innovation. It is probable that the implementation of creative ideas, products, or procedures may require additional activities beyond the control of the individual worker subject to input, process, and output constraints such as rules and regulations, deadlines, and resources (refer to Acar et al., 2019). Alternatively, the implementation of creative ideas may require a longer timeframe to manifest than the 4-week timeframe of the data collection for this study.

Although individual-perceived PSC predicted future work performance, at the between-person level, both incremental and radical creativity were not related to work performance, and creativity did not mediate the relationship between PSC and future work performance. These results support other findings challenging the assumption that creative work increases general work performance (e.g., Mumford et al., 2002; Gilson, 2008; Zhou and Shalley, 2008). The complex work of software engineering performance is likely to involve duties and responsibilities beyond creative activities, or there may be additional unexplored moderators of the creativity–work performance relationship, such as the level of work experience impacting the employees' ability to effectively manage competing creative and routine performance demands (Zhang and Bartol, 2010).

### Theoretical development

This is the first known study to take a dynamic approach to examine how the climate for psychological health, PSC, influences creative processes *in situ* over time as they naturally unfold using short weekly time lags with a longitudinal multilevel research design. Extending the existing PSC literature, which has focused predominantly on the relationship between PSC and psychological health problems, we found that the influence of PSC may also be important in boosting creative work processes. The findings suggest that workers in a high PSC context who are not fighting to maintain depleted psychological resources (Brunetto et al., 2022) will have a greater capacity to undertake creative tasks involving handling task-related pressure (Taylor et al., 2019), investing energy to maintain a task goal in memory, employing efficient cognitive search strategies (Dollard et al., 2019), and refining ideas leading to higher levels of radical and incremental creative thinking. Alternatively, creative behavior may be a reciprocal response (social exchange theory, Blau, 1964) to the feeling of having one's psychological health valued and protected within the organization. Most theories of creativity have focused on individual and group creative support systems with stimuli provided to increase employee creative thinking and behavior (e.g., cognitive network model of creativity, Santanen et al., 2004; bisociation theory, Koestler, 1964; dynamic stimuli, Knoll and Horton, 2011; Wang and Ohsawa, 2013; Althuizen and Wierenga, 2014) which are proposed to have varying influence depending upon the cognitive style of the employee (Nagasundaram and Bostrom, 1994; Garfield et al., 2001; Madjar and Oldham, 2006; Cheung and Chau, 2008; Althuizen and Wierenga, 2014; Wang and Nickerson, 2017). This study expands these theoretical conceptualizations suggesting that the perceived climate for psychological health and safety, PSC, may be a multilevel predictor and a broad contextual generator of these individual cognitive, creative processes.

The findings add to the climate literature for creativity and innovation and take initial steps to address a gap in the literature highlighted by Newman et al. (2020) to contribute more longitudinal multilevel empirical work to identify whether climates for innovation and creativity (e.g., Climate for Innovation Scale, Scott and Bruce, 1994; Team Climate Inventory (TCI), Anderson and West, 1996, 1998) perceived by individuals predict similar or different creative outcomes to alternative climates, such as a climate for safety (e.g., psychological health and safety, PSC, Dollard and Bakker, 2010: synergistic diversity climates, Richard et al., 2019). While climates for creativity have been examined in previous research (Mathisen and Einarsen, 2004; Mathisen et al., 2004, 2008; West and Richter, 2008; Sung et al., 2018) with a strong emphasis on factors in the work environment that increase or reduce creativity or innovation, the influence of a climate for the support of psychological health on creativity in digital work has not been examined. Our findings suggest a new theoretical conceptualization of the development of creative ideas through the lens of the protection of psychological health, where creativity arises through freedom from psychosocial risks leading to additional resourcing for creative energy and thought. This conceptualization contrasts with other theories related to creative and innovation climates, which predominantly focus on motivational processes where creative and innovative climates increase intrinsic and/or extrinsic motivation to complete creative tasks (Zhu et al., 2018; Newman et al., 2020).

Scholars have raised the need to differentiate radical and incremental creativity (Madjar et al., 2011; Malik et al., 2019). According to the current literature on incremental and radical creativity, they are precipitated by different underlying processes (Gilson and Madjar, 2011; Madjar et al., 2011; Gilson et al., 2012; Liu et al., 2021). This may be particularly salient in digital work because radical creativity may be more difficult to perform in a computer-based environment where there may not be spontaneous social interactions to alleviate high risks and social rejection associated with the collective exchange of novel ideas (DeRosa et al., 2007; Sung et al., 2020). Notably, PSC predicted future radical and incremental creativity. When the engagement was entered into the multilevel model, PSC continued to account for significant variance in radical creativity only. This suggests that PSC may have a stronger influence on radical creativity than incremental creativity. Radical creativity requires cognitive persistence, learning goal orientation, the ability and resources to engage in problem-driven processes, and a willingness to take risks requiring an investment of greater cognitive resources (Gilson and Madjar, 2011; Madjar et al., 2011; Malik et al., 2019). Generating novel radical ideas may require strong executive functioning, including working memory, inhibition, and fluid intelligence. Poor psychosocial work environments (low PSC) characterized by high levels of work stress likely to compromise the cognitive resources available to the employee for radical creative thought (e.g., Amabile et al., 1990; Oldham and Cummings, 1996; Shalley and Perry-Smith, 2001). This greater investment of resources may require a stronger climate for psychological health and safety (high PSC) compared to incremental creative processes, which engage solution-driven processes within the confines of existing practices (Gilson and Madjar, 2011; Madjar et al., 2011; Malik et al., 2019). Further research may elucidate the mechanisms involved.

### Practical implications

Although the strength of these relationships is currently limited due to the restricted power of the study, initial findings suggest that creativity may be enhanced in high PSC work environments. If senior managers seek to improve individual levels of incremental and radical creativity in dynamic complex computer-based project work environments, then targeting improvement in PSC may foster conditions to trigger and support individual creative activities. Managers can measure and initiate evidence-based workplace approaches to improve the PSC using established tools (Hall et al., 2010; Dollard, 2019), benchmarks (Bailey et al., 2015), and action-planning strategies (refer to Bailey et al., 2014; Dollard and Gordon, 2014; Dollard et al., 2016; Loh et al., 2020; Dollard and Bailey, 2021).

### Limitations and future research

While the study developed a good understanding of the underlying relationships, extending the sample size and the number of weeks surveyed may increase our capacity to detect true effects. We note that for week-level diary studies, the number of weeks usually ranges between 3 and 5 weeks, thus, our 4 consecutive week timeframe for this study is consistent with the current recommended practice (Bakker and Bal, 2010; Bakker and Sanz-Vergel, 2013). Due to privacy concerns initiated by the organization, the study did not control for demographics or work status (i.e., position or tenure). However, it is argued that these issues may not affect the variances within an individual (Podsakoff et al., 2019). We also acknowledge that the small sample size in this current study might lead to low statistical power; however, a range of authors suggest that for a multilevel model, the minimal L2 number is 30 for a cross-sectional fixed effect (which is the effect in the current study) which is close to our sample size (see Maas and Hox, 2005; Snijders, 2005; McNeish and Stapleton, 2016; Hox and McNeish, 2020). In addition, we reported the results with robust standard errors, which may provide a more accurate result.

Self-report data have often been criticized due to the construct validity of self-reported perceptions; however, many studies have demonstrated the criterion-related validities of self-report predictor measures (Chan, 2010). Potential common method variance and socially desirable responding generated by self-report measures were addressed in accordance with recommended practice using the temporal separation of measurement of independent and dependent variables across a 4-week period, by protecting the anonymity of the respondents and by using psychometrically validated scales (Chan, 2010; MacKenzie and Podsakoff, 2012; Podsakoff et al., 2012; Tehseen et al., 2017). Chan (2010) indicates that the use of self-report measures is not only justifiable but also needed when assessing constructs involving self-referential respondent perceptions such as creativity, innovation, and work performance involving subtle individual nuance across dynamic complex environments. While the possibility of method bias and its limitations to confirm causal effects exists, self-report data remain useful as a method to capture employees' attitudes, perceptions, and behaviors (Organ and Konovsky, 1989; Janssen, 2000; Chan, 2010) and informative as an explorative study. Our study is among the first initiatives investigating the link between PSC and perceived fluctuations in creativity hence providing some insights into the influence of PSC on employees' perceived behavior.

Future research could consider the automated assessment of creativity such as a stealth assessment or the influence of PSC on the use of virtual tools to diagnostically assess or support workers' creativity (Guegan et al., 2016; Shute and Rahimi, 2021). Supervisor ratings of creative work performance may increase the relative objectivity of the rating; however, these may also be limited as they are only able to report on visible manifestations of creativity when the creative process involves many internal psychological processes as well (Op den Kamp et al., 2022). Future research could examine the influence of PSC on collaborative processes among employees including virtual and face-to-face group interactions when working on creative idea generation tasks, a process dubbed “group brainstorming”; meta-analytic data have identified creative advantages for virtual groups such as the anonymity present in electronic interaction, inherent memory advantage, and increased creative productivity due to the presence of visual information (DeRosa et al., 2007). Notably, increased creative fluency and a higher number of unique ideas have been seen when social identify cues are used in virtual and face-to-face group settings suggesting that a sense of belonging may stimulate creativity which may have the potential to be explored in the climate level (Guegan et al., 2017). Recent research has identified that leader-member exchange and team-member exchange (Pan et al., 2012; Seong and Choi, 2019) and employee appraisals of creative performance pressure as challenging or hindering (Liu et al., 2022) influence subsequent creative output. Status differential and participation in knowledge-sharing processes may also impact creativity (Sung and Choi, 2019). Examining the role of the climate for psychological health (PSC) as a predictor and moderator of these relationships would identify how climates impact these psychosocial antecedents of creativity. Recent meta-analytic research also indicates that perceptions of the organizational context, such as the perceived PSC, may differentially impact personality types such as Openness, Extroversion, and Conscientiousness, and interactions could be examined in more detail (Zare and Flinchbaugh, 2019).

## Conclusion

Computer-based environments need to foster creativity for competitive advantage. This study identifies a gap in current creativity research identifying how the climate for worker psychological health (PSC) stimulates the growth of workplace creativity and performance in digital environments. In a software engineering environment, individuals reporting high PSC had higher future weekly individual fluctuations of digital creativity, work engagement, and job performance. Work engagement also mediated the relationship between PSC and future creativity. The findings propose that PSC influences creativity in digital environments suggesting that prioritizing a climate for psychological health and safety will likely enhance future digital creativity.

## Data availability statement

The raw data supporting the conclusions of this article will be made available by the authors, without undue reservation.

## Ethics statement

The studies involving human participants were reviewed and approved by University of South Australia Human Research Ethics Committee Protocol no.: 202172. The participants provided their informed consent to participate in this study.

## Author contributions

AZ conceptualized the project and completed the literature review, data collection and initial preliminary statistical analyses, and wrote the initial draft of the paper. ML completed the statistical analysis and assisted with the theoretical development and writing of the article. MD contributed to the development of the project, survey design, theoretical conceptualization, and writing of the article. GM contributed to the literature review, theoretical conceptualization, and writing of the article. BY contributed to the development of the project, survey design, and writing of the article. All authors contributed to the article and approved the submitted version.
